# Development of the Subjective Cognitive Decline Scale for Mandarin-Speaking Population

**DOI:** 10.1177/15333175211038237

**Published:** 2021-09-07

**Authors:** Hsing-Fang Tsai, Chi-Hsun Wu, Chih-Cheng Hsu, Chien-Liang Liu, Yen-Hsuan Hsu

**Affiliations:** 1 Clinical Psychology Center, 38006 National Taiwan University Hospital, Taipei, Taiwan; 2 Department of Psychology, 34913 National Chengchi University, Taipei, Taiwan; 3 Institute of Population Health Sciences, 50115 National Health Research Institutes, Zhunan, Miaoli County, Taiwan; 4 Department of Neurology, 38010 Taipei City Hospital, Taipei, Taiwan; 5 Department of Psychology, 34915 National Chung Cheng University, Chiayi County, Taiwan; 6 Center for Innovative Research on Aging Society (CIRAS), 34915 National Chung Cheng University, Chiayi, Taiwan

**Keywords:** subjective memory complaints, dementia, cognitive complaints, questionnaire, self-report, subjective cognitive decline, mild cognitive impairment

## Abstract

Subjective cognitive decline (SCD) has been considered a high-risk group preceding mild cognitive impairment (MCI). However, methods to quantify and track the complaints have not been well-established. The present study aimed to develop a questionnaire tailored for Mandarin-speaking individuals with SCD. A total of 175 adults aged above 55 years completed a comprehensive set of items evaluating cognitive problems and neuropsychological examinations. After item reduction, internal consistency, construct, and concurrent validity were examined. The 14-item Subjective Cognitive Decline Scale (SCDS) has acceptable internal consistency (Cronbach’s *α* = .93) and construct validity with a three-factor structure. Individuals with SCD and MCI scored higher than the control group. The SCDS demonstrated significant but small correlations with multiple cognitive tests and emotional variables. The SCDS provides an alternative approach to measure cognitive complaints, while an influence of emotional status shall be taken into consideration when interpreting the results.

## Significance Statement

• The SCDS has three factors that evaluate memory, executive function, and language problems.
• The SCDS showed proper psychometric properties for documenting subjective cognitive decline in Mandarin-speaking older adults.

## Introduction

Subjective cognitive complaint is common among older adults with or without mild cognitive impairment (MCI).^1^ Meanwhile, the person with subjective cognitive complaint may still perform normally on neuropsychological tests^2,3^ as a result of behavioral/neural compensation or high premorbid function against the normative comparison standards. This status is referred to as subjective cognitive decline (SCD).

Despite an association with emotional status,^4^ recent studies showed that SCD indicated an elevated risk of subsequent cognitive deterioration,^5,6^ which constitutes a clinically identifiable stage preceding MCI.^2^ Increasingly, researchers advocate the importance of SCD as it could provide information for screening, monitoring, treatment, and researching.^7^

However, standardized measures to quantify SCD have been limited. Some studies utilized a single yes/no question to classify the presence of SCD, and some adopted questionnaires that concentrated on memory function.^5,8^ Moreover, many existing questionnaires (Table 1) were not designed for individuals with SCD, had diverse content coverage, inconsistent reference periods for answers, and were developed predominantly in English-speaking population.^9^

**Table 1.** Summary of Questionnaires Used in SCD Studies.

	Authors and years	Source of Report	No. Of Item	Reference Point	Response Type	Dimensions	Tested Population	Psychometric Data	Area
Cognitive Failures Questionnaire (CFQ)	Broadbent et al^47^	Self- and informant-report	25 for self-report; 8 for informants’ report	In the last 6 months	Five-point Likert scale (from “never” to “very often”)	Unidimensional, items covering perceptual, memory, and action failure	Students, workers, and managers	Internal consistency; construct validity; external validity	United States
Everyday Cognition (ECog)	Farias et al^48^	Informant	39	10 years ago	Four-point Likert scale (from “better or no change” to “consistently much worse”)	Global factor and domain-specific factors (memory, language, visuospatial, planning, organization, and divided attention)	Normal cognition, MCI, or dementia	Test–retest reliability; content validity; construct validity; external validity	United States
Memory Complaint Questionnaire (MAC-Q)	Crook et al^32^	Self-report	6	High-school or college years	Five-point Likert scale (from “much better” to “much poorer”)	Memory	Age-associated memory impairment^*^ (aged above 50)	Test–retest reliability; internal consistency; concurrent validity	United States
Memory Functioning Questionnaire (MFQ)	Gilewski et al^33^	Self-report	64	59 items of current function; 5 items comparing memory to 1, 5, 10, and 20 years ago and at the age of 18	Seven-point Likert scale (from “always” to “never,” from “very good” to “very bad,” from “not serious” to “very serious,” and from “much better” to “much worse”)	General frequency of forgetting; seriousness of forgetting; retrospective functioning; and mnemonics usage	Adults from university, community college, community, and health maintenance organization (aged 16 to 89)	Internal consistency; construct validity	United States
Subjective Cognitive Complaints (SCCs) index	Slavin et al^36^	Self- and informant-report	24 for self-report; 19 for informants’ report	5 and 10 years ago	Yes or no	Memory and non-memory	Individuals with MMSE ≥24 (aged 70 to 90)	Construct validity; external validity	Australia
Subjective Cognitive Decline Questionnaire (SCD-Q)	Rami et al^42^	Self- and informant-report	3 global and 24 specific items	2 years ago	Yes or no	Unidimensional, items covering memory, language, and executive function	Cognitively healthy controls, SCD, MCI, and AD patients (aged above 45)	Internal consistency; content validity; construct validity; concurrent validity; external validity	Spain
Subjective Memory Scale (*SMS*)	Snitz et al^45^	Self-report	2 global items and 14 specific items	Current function; 1 year ago	Four-point Likert scale (from “excellent” to “poor”); three-point Likert scale (from “better” to “worse”); yes or no (“remember less well,” “worried about the problem,” and “had talked to doctor”)	Memory	Primary care patients without dementia diagnosis (aged above 65)	Criterion validity	United States

^*^Age-associated memory impairment: performance on standardized memory tests at least one standard deviation below the mean for young normal adults.

Therefore, the present study aimed to develop a questionnaire tailored for individuals with SCD in Mandarin-speaking population and to examine its psychometric properties with a cohort of non-demented older adults.

## Methods

### Participants

**Figure 1.**
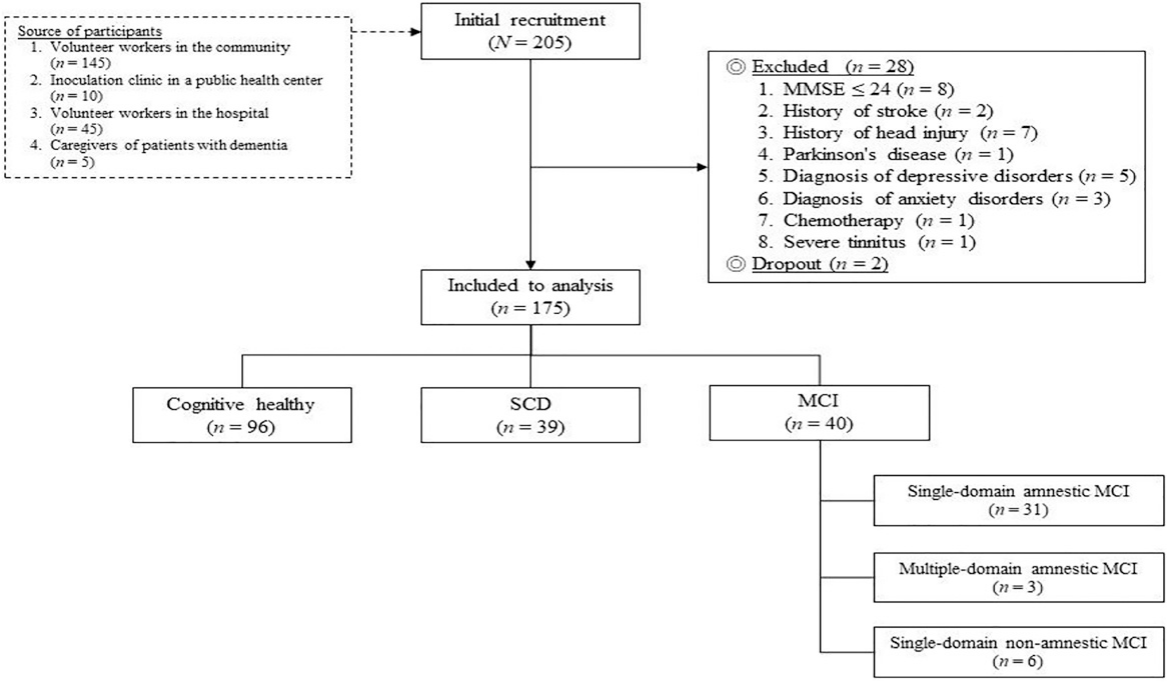
Baseline recruitment process.

Eligible participants were 205 non-demented adults older than 55 years recruited from the communities from August 2017 to January 2018 through advertisement and snowball sampling (Figure 1). All participants were fluent in Mandarin. Exclusion criteria included diagnosis of dementia and other psychiatric (eg, depression, anxiety, and schizophrenia, etc.) or neurological illness (eg, stroke, head injury, movement disorders, etc.); other systematic illness, medication, or substance use that may affect cognitive function; severe visual, hearing, or communication disturbance; other causes that prohibit cooperation. A total of 175 participants were included in the analysis (Table 2). All participants signed on written informed consent before entering the study, and the study protocol was approved by the institutes’ committee on human research (TCHIRB-10605109-E and EC1060512).

**Table 2.** Basic Information of the Participants in the First-Wave Assessment (N = 175).

	SCD (N = 39)		MCI (N = 40)		CN (N = 96)		*F* or *χ*^*2*^	*p*
	Mean	*SD*	Mean	*SD*	Mean	*SD*		
Age (years)	66.97	6.10	65.92	6.07	65.10	5.28	1.55	.21
Sex (men: women)	11: 28		11: 29		18: 78		2.04	.36
Education (years)	12.77	3.62	11.25	3.66	13.42	3.02	6.05	.003^c^
Emotional status								
BDI	5.41	4.72	3.07	4.22	3.99	4.92	2.88	.06
BAI	3.33	3.43	2.78	4.15	2.62	3.47	.62	.54
T-PSWQ	37.54	10.95	34.90	9.65	32.59	9.32	5.00	.008^a^
Cognitive function								
MMSE	28.72	1.45	27.95	1.41	28.95	1.09	4.55	.01
CVVLT								
Immediate recall	28.08	3.16	22.60	2.98	28.65	3.10	47.04	<.001^a,c^
Short-delay recall	7.77	1.25	5.98	1.39	8.06	.97	39.72	<.001^a,c^
Long-delay recall	7.13	1.98	5.55	1.50	7.77	1.20	26.48	<.001^a,c^
Cued recall	7.62	1.58	5.83	1.68	7.95	1.22	28.43	<.001^a,c^
Recognition	8.69	.57	7.80	1.47	8.72	.60	14.78	<.001^a,c^
Verbal fluency	11.74	3.19	10.08	3.48	11.79	3.25	1.99	.14
Digit Span								
Forward	11.56	2.09	11.33	2.37	12.30	2.36	1.62	.20
Backward	7.08	2.34	5.88	2.12	7.23	2.54	2.10	.13
Sequencing	8.33	1.98	7.48	1.55	9.02	2.10	5.19	.006
Digit Symbol Coding	61.13	15.09	58.07	16.12	64.65	14.95	.67	.51
CTT 1 (seconds)	53.38	16.16	55.65	18.37	50.55	17.38	.17	.84
CTT 2 (seconds)	107.49	26.79	121.55	42.55	103.01	31.75	2.41	.09
Judgment of Line Orientation	20.00	4.33	18.65	4.39	19.90	4.21	.67	.51
Taiwanese Frontal Assessment Battery	13.92	2.44	13.20	2.39	15.03	1.90	6.23	.002^c^
Prospective memory score	6.13	1.88	5.93	1.94	6.30	1.66	.29	.75

^a^Significant difference between the SCD and the MCI groups.

^b^Significant difference between the SCD and the CN groups.

^c^Significant difference between the MCI and the CN groups.

Abbreviations: MCI, mild cognitive impairment; SCD; subjective cognitive decline; CVVLT, Chinese version verbal learning test; MMSE, Mini-Mental State Examination; T-PSWQ, Taiwanese version of the Penn State Worry Questionnaire; CN, cognitively normal; BAI, beck anxiety inventory.

Participants were further categorized into three groups: the MCI, the SCD, and the cognitively normal (CN). The diagnostic criteria for MCI were based on the comprehensive criteria proposed by Jak et al^10^ that were shown to have a good diagnostic reliability. Participants with at least two test scores worse than 1 standard deviation (*SD*) below means of age- and education-corrected normative data within at least one cognitive domain were assigned to the MCI group. Participants who did not fulfill the criteria of MCI but reported current memory or cognitive difficulty were categorized into the SCD group. Participants who did not fulfill the criteria of MCI and reported no current memory or cognitive difficulty were categorized into the CN group. All participants had a score not more than nine on the Functional Activities Questionnaire.^11^

### Procedure

#### Item construction

Seven questionnaires with test items available online were identified by literature review (Table 1). Items were first selected based on the occurrence frequency across questionnaires and expert review of the representativeness for its category. The resulting 50 items evaluate orientation (three items), attention (four items), memory (23 items), language (six items), visuospatial (four items), and executive function (10 items).

The response option was a five-point Likert scale (from “strongly agreed” to “strongly disagreed”). In this initial item set, an additional “not applicable” response was available to evaluate age and cultural appropriateness for each item. The reference point for response was set at one year ago, based on suggestions from previous studies.^9,12^ All written materials were presented in Traditional Chinese characters.

### Neuropsychological Tests

A semi-structured interview was first conducted to collect basic information. In order not to affect the participants’ subjective belief, self-reports were given prior to neuropsychological tests. Neuropsychological test scores used to classify participants included the immediate recall and the long delay recall scores on the Chinese Version Verbal Learning Test (CVVLT)^13^; forward, backward, and sequencing scores of the Digit Span subtest, and the total score of the Digit Symbol Coding subtest from the Taiwanese version of the Wechsler Adult Intelligence Scale–Fourth Edition^14^; the completion time on the Color Trails Test (CTT) 1 and 2^15,16^; and the total score of the Taiwanese Frontal Assessment Battery (TFAB).^17,18^

Additional cognitive tests were included for examining concurrent validity. The Taiwanese version of the Mini-Mental State Examination MMSE^19,20^ was used to evaluate general cognitive function. The Judgment of Line Orientation^21,22^ was performed to assess visuospatial function. The Semantic Association of Verbal Fluency^23,24^ was used to reflect language function. Two single-trial prospective memory tasks were also used to rate event-based and time-based prospective memory function.^25^

To control for the influence of emotional variables, the Beck Depression Inventory,^26^ the Beck Anxiety Inventory,^27^ and the Taiwanese version of the Penn State Worry Questionnaire T-PSWQ^28^ were used to assess the participants’ emotional status.

### Statistical Analysis

Demographic variables, neuropsychological performance, and emotional status were compared between the SCD, MCI, and CN groups using analysis of variance (ANOVA) or analysis of covariance (ANCOVA), where appropriate. Significant results were followed by *post hoc* analysis with Bonferroni tests. Type I error *α* was set at .01 to control for familywise error rate.

Item reduction was performed based on the following criteria: (1) missing data more than 10% (“not applicable” answer), (2) means over 4.5 or lower than 1.5, (3) variance lower than 1, (4) absolute value of skewness more than 1, (5) insignificant results from independent *t*-tests (critical ratio) when comparing performance between the high- and low-score groups, (6) correlation with the full scale less than .3, and (7) an elevated Cronbach’s *α* if item deleted. Subsequently, exploratory factor analyses (EFAs) were conducted and items were further removed based on communalities and factor loadings. Missing values *(“*not applicable” answer) were treated with expectation maximization. Factor numbers were decided by parallel analysis.^29^ Principal axis factoring with promax rotation was used as the factor extraction method. Cronbach’s *α* was used to examine the remaining items for internal consistency.

Regarding construct validity, confirmatory factor analysis (CFA) was performed with AMOS^30^ using 2000 samples from bootstrapping resampling and maximum likelihood estimation. Seven indices were used to examine model fit, including the chi-square (*χ*^2^), the normed chi-square (*χ*^2^/df), the root-mean-square error of approximation (RMSEA), standardized root-mean-square residual (SRMR), the goodness-of-fit index (GFI), the Tucker–Lewis index (TLI), and the comparative fit index (CFI).

Concurrent validity was examined by correlational analyses between the SCDS scores and neuropsychological test results. Partial correlation was performed to tease out potential influence of age and education. Discriminant validity was examined by correlations between subscales and cognitive tests that measure different functions and between subscales and emotional status. Construct validity was evaluated by comparing the SCDS scores reported by the SCD, MCI, and CN groups using ANCOVA. *Post hoc* analysis was carried out with Bonferroni tests (*α* set at .05).

## Results

### Characteristics of the Participants

There was significant group difference of education (*P* = .003) and T-PSWQ scores (*P* = .008), in which the MCI group was less educated than the CN group (*P* < .05) and scored less on the T-PSWQ (*P* < .05). There was no significant difference between groups on age, sex, BDI-II, or BAI scores (Table 2). Thus, education and T-PSWQ were used as covariates in the subsequent group mean comparisons. Regarding neuropsychological performances, the MCI group performed significantly worse than the CN group on multiple memory indices of the CVVLT (*P* < .001) and the TFAB (*P* = .002); the MCI groups also performed significantly worse than the SCD group on the CVVLT (*P* < .001).

### Item Reduction

The results of initial review of the 50 test items indicate removal of items 5, 7, 14, 20, 24, and 25 according to the criteria described above. Another two items (36 and 45) were also removed due to low response variance and critical ratio, respectively.

EFA was conducted with the rest of the items. The Kaiser–Meyer–Olkin (KMO) test for sampling adequacy resulted in a value of .94, and the Bartlett’s test of sphericity was significant (*χ*^2^ = 5982.81, *P* < .001), indicating that the data were appropriate for factor analysis. Parallel analysis suggested extracting three factors. Principal axis analysis with promax rotation showed that items 2, 9, 18, 30, 33, 37, and 38 had factor loading lower than .4. These items were removed to retain construct validity. The second EFA with the rest of the items showed that factor 2 mainly reflected memory function except from item 10, which was then removed. Items reflecting memory function but categorized into factor 1 and factor 3 were also removed, including items 3, 6, 8, 17, 29 31, 32, 40, 44, and 48. The third EFA with the rest of the items showed that factor 3 mainly reflected language function. Thus, items that measure language function but categorized into factor 1 were removed, including items 16 and 46. The third EFA showed that factor 1 was mainly constituted of items assessing executive function and orientation. Item 22 that evaluates executive function was categorized into factor 3 and was removed. The remaining 22 items were used for another EFA, which indicates a three-factor structure. Subsequently, multiple EFAs were performed where the test item with the lowest factor loading was removed in each trial. Items 4, 12, 15, 19, 21, 41, and 42 were removed considering the explained variance each of them contributed. The final version of the SCDS contains 14 items (Supplementary appendix A1), and EFA showed a three-factor structure (Table 3). Factor 1 comprises six items that assess executive function and orientation (the Executive function subscale, SCDS-Exe); factor 2 contains four items evaluating memory function (the Memory subscale, SCD-Mem); and factor 3 is consisted of four items concerning language function (the Language subscale, SCDS-Lang). The three factors explained 60.48% of the variance. The relationship was *r* = .64 between factor 1 and factor 2, *r* = .75 between factor 1 and factor 3, and *r* = .64 between factor 2 and factor 3.

**Table 3.** Results of the Final EFA for the SCDS.

Items^*^	Factor		
	1	2	3
**#E-23**	**.768**	.155	-.177
**#O-35**	**.752**	-.013	-.006
**#O-34**	**.737**	-.113	.187
**#E-11**	**.661**	.076	.124
**#O-01**	**.646**	-.073	.136
**#E-13**	**.610**	.171	.066
**#M-49**	.078	**.823**	-.077
**#A-50**	-.115	**.799**	.058
**#M-43**	.065	**.587**	.078
**#A-47**	.137	**.522**	.133
**#L-28**	.026	.010	**.895**
**#L-27**	.098	.064	**.694**
**#L-26**	-.106	.219	**.692**
**#L-39**	.283	-.123	**.600**

^*^Items were listed by the number and category in the initial item set (E: Executive function; O: Orientation; M: Memory: A: Attention; L: Language); Bold font indicates factor loading larger than .4.

Abbreviations: EFAs, exploratory factor analyses; SCDS; subjective cognitive decline scale.

### Internal Consistency

The SCDS showed good internal consistency (Cronbach’s *α* = .93). The three subscales also demonstrated proper internal consistency (SCDS-Exe: Cronbach’s *α* = .89; SCDS-Mem: Cronbach’s *α* = .83; SCDS-Lang: Cronbach’s *α* = .88).

### Construct Validity

**Figure 2.**
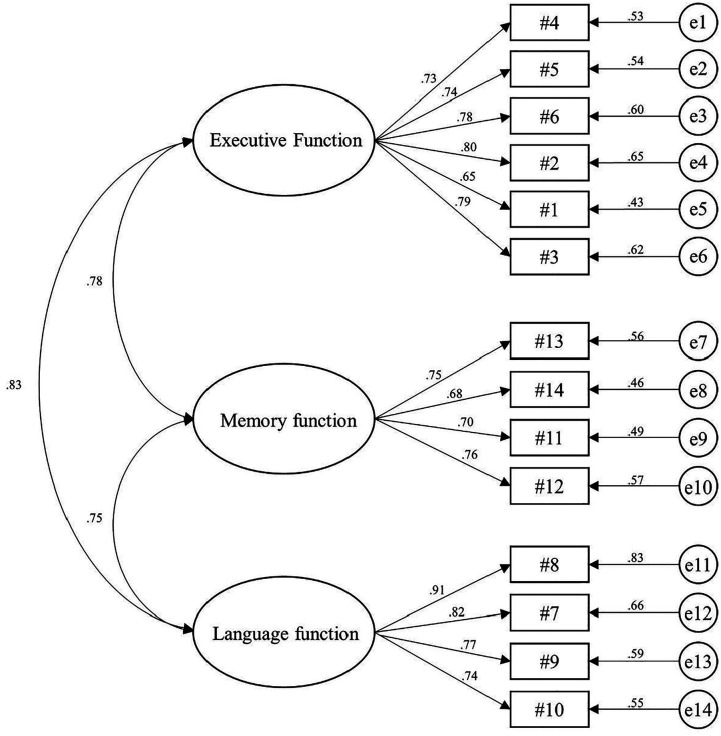
Factor structure of the Subjective Cognitive Decline Scale.

The three-factor model of the self-reported SCDS was examined by CFA with bootstrap resampling (Figure 2). The results showed that all factor loadings were acceptable, ranging from .5 to .95. Although the chi-square test yielded a significant result (*χ*^2^ = 147.15, *P* < .001), the normed chi-square (*χ*^2^/df = 2.07) and all other five indices showed acceptable level of model fit (RMSEA = .08; SRMR = .04; GFI = .91; TLI = .93; CFI = .95). Regarding convergent validity, the Composite Reliability (CR) and Average Variance Extracted (AVE) values were above .60 and .50, respectively, among all factors. Analyses of correlations between factors showed that all pairs had their values with the confidence intervals less than 1, showing adequate discriminant validity.

### Concurrent Validity

After controlling for age and education, the SCDS total scores were significantly correlated with the MMSE scores (*r* = −.18, *P* = .03; Table 4). Significant correlations were also found between the SCDS-Exe scores and tests of executive function, including the CTT2 (*r* = .17, *P* = .04) and TFAB (*r* = −.21, *P* = .01), and between the SCD-Mem scores and the CVVLT long-delay recall scores (*r* = −.18, P = .03). There was no significant correlation between the SCDS scores and performance on the Judgment of Line Orientation that assesses visuospatial function (*P* > .48).

**Table 4.** Correlations Between the SCDS-Q and Cognitive Test Performances (N = 175).

	SCDS-Q (Total Score)	SCDS-Exe	SCDS-Mem	SCDS-Lang
Global cognitive function				
MMSE	−.180^*^	−.213^**^	−.104	−.135
Executive function				
CTT2 (seconds)	.175^*^	.171^*^	.200^*^	.083
TFAB	−.208^*^	−.209^*^	−.168^*^	−.163^*^
Prospective memory score	.044	−.003	.065	.072
Memory function				
CVVLT immediate recall	−.103	−.111	−.023	−.131
CVVLT long-delay recall	−.242^**^	−.252^**^	−.177^*^	−.198^*^
Language function				
Verbal fluency	−.037	−.024	−.038	−.039
Visuospatial function				
Judgment of Line Orientation	−.017	−.008	.016	−.056
*Emotional status*				
BDI	.349^***^	.294^***^	.261^***^	.379^***^
BAI	.383^***^	.359^***^	.277^***^	.375^***^
T-PSWQ	.380^***^	.368^***^	.343^***^	.282^***^

^*^P < .05; ^**^P < .01; ^***^P < .001.

However, the SCDS-Lang was not correlated with language performance on verbal fluency (*r* = −.04, P = .63). The SCDS-Exe scores were also found to correlate with the CVVLT long-delay recall (*r* = −.25, P = .002), the SCD-Mem scores were associated with the CTT2 (*r* = .20, P = .01) and TFAB (*r* = −.17, P = .04), and the SCDS-Lang was correlated with the TFAB (*r* = −.16, P = .04) and the CVVLT long-delay recall (*r* = −.20, P = .01).

Furthermore, all SCDS subscale scores were significantly associated with scores on BDI (*r* = .26 ∼ .38), BAI (*r* = .28 ∼ .38), and T-PSWQ (*r* = .28 ∼ .38).

### SCDS Scores Between Groups

There was a significant difference on the SCDS score between the three groups even after controlling for emotional disturbance (*F* = 6.05, P = .003, *η*_*p*_^*2*^ = .07; Table 5). *Post hoc* tests showed that the CN group had significantly lower SCDS scores than the SCD and MCI groups (*P* < .05). Regarding scores on each subscale, significant group difference was found on the SCDS-Exe (*F* = 4.26, P = .016, *η*_*p*_^*2*^ = .048) and SCDS-Mem (*F* = 8.01, *P* < .001, *η*_*p*_^*2*^ = .086) subscales. A trend of group difference was observed on the SCDS-Lang (*F* = 3.02, P = .052) subscale. *Post hoc* analysis showed that the SCD and MCI groups reported significantly more memory problems than the CN group on the SCDS-Mem subscale (*P* < .05). The SCD group also reported significantly more problems on the SCDS-Exe subscale than the CN group (*P* < .05).

**Table 5.** Comparisons of the SCDS Scores Between the SCD, the MCI, and the CN Groups.

	SCD			MCI			CN			ANCOVA^†^	
	N	*M*	*SD*	N	*M*	*SD*	N	*M*	*SD*	*F*	*p*
SCDS											
Executive function subscale (6 items)	39	13.68	4.82	40	13.20	4.39	96	10.95	3.99	4.26^b^	.016
Memory function subscale (4 items)	39	10.51	3.32	40	10.23	3.56	96	8.05	2.49	8.01^b,c^	<.001
Language function subscale (4 items)	39	8.67	3.21	40	9.29	3.07	96	7.52	2.93	3.02	.052
Total score (14 items)	39	32.86	9.53	40	32.73	9.34	96	26.52	8.61	6.05^b,c^	.003

^†^Education and T-PSWQ as covariates.

Abbreviations: MCI, mild cognitive impairment; SCD; subjective cognitive decline; CN, cognitively normal; ANCOVA: analysis of covariance.

^a^Significant difference between the SCD and the MCI groups.

^b^Significant difference between the SCD and the CN groups.

^c^Significant difference between the MCI and the CN groups.

## Discussion

The present study aimed to develop a quantitative method designed for measuring SCD and examine its psychometric properties. To the best of our knowledge, this is the first attempt to develop a questionnaire for this purpose in Mandarin-speaking population. The resulting 14-item SCDS has three subscales, including the Memory (SCDS-Mem), Executive Function (SCDS-EXE), and Language (SCDS-Lang) subscales. The SCDS demonstrated good internal consistency, construct validity, and correlations with multiple cognitive test results. However, the ratings were also moderately associated with emotional status.

Test instruments in the format of self-report are relatively simple to administer and versatile in its content. As binary response format in a single test item (e.g., “Do you feel like your memory is becoming worse?“) does not readily capture the severity and specificity of cognitive problems, a growing body of research adopted cognitive questionnaires to evaluate SCD.^31–37^ When assessing subjective memory impairment, it is important to specify a length of time, to provide a valid example, and to rate the frequency of problems.^38^ Since there has been no consensus regarding which questionnaire should be used for SCD, the present study reviewed test items from published questionnaires in SCD research, adjusted the response format and reference period, and examined psychometric properties of the newly developed SCDS.

The three-factor SCDS captures cognitive problems in memory, executive function, and language domains. The SCD-Mem subscale was shown to best differentiate between the SCD and controls, indicating sensitivity to subtle memory change. The SCD-Exe subscale not only included test items of executive function but also items that would traditionally be categorized into temporal orientation. Sometimes considered a sign of memory loss,^39^ temporal disorientation has also been associated with impairment of executive function.^40^ The SCDS-Lang subscale included items assessing abilities of verbal comprehension, word-finding, describing a plot, and initiating a conversation. The SCDS did not include items evaluating visuospatial function due to high percentage of “not applicable” response or inadequate difficulty in the initial data set. For example, some items concern the ability to use a map or to find a car. A sizable portion of participants reported difficulty due to visual impairment or lack of opportunity in real life, and others reported never learn to drive or not having a car. Further study may consider listing better descriptions for everyday visuospatial problems. Overall, the self-reported SCDS did not show a factor structure corresponding to the original item categorization, but was partly similar to the four-factor structure of SCD-Q.^41^ Clarification of cognitive problems into six categories may be elusive for non-professionals raters.

It is noteworthy that the SCDS scores were close between the SCD and MCI groups, which is in line with previous report^42^ and partially supports the working model^43^ that subjective cognitive complaints gradually increase as underlying pathology progresses, achieving the peak at late SCD and early MCI before receding due to emerging anosognosia.

In accordance with previous studies,^44,45^ significant but small correlations were found between SCDS scores and cognitive performances. The misalignment between subjective and objective cognitive measures may result from issues of test sensitivity, specificity, intra-individual performance-variability, inter-rater reliability, and cognitive compensation.^3^ In addition, the lack of variation in language abilities at baseline in our sample may partly contribute to the lack of correlations between self-reported and performance-based language measures. On the other hand, small to moderate positive relationships were observed between subjective reports of cognitive and emotional status, even after excluding individuals with diagnosis of depressive or anxiety disorders. Similar finding has been reported in the past.^42^ Of note, the SCD group in our study did report higher level of worries than the controls. Perrotin et al^46^ pointed out that SCD could be associated with elevated level of anxiety and amyloidosis. However, it was suggested not excluding mild emotional symptoms in SCD research since emotional disturbance also occurs in early stages of neurodegenerative disease.^12^

One of the contributions of the present study was to develop a questionnaire that quantifies SCD in a group that was linguistically and culturally different from those in previous studies (Table 1). Several test items that had been used in SCD studies were removed. For example, 13.1% to 23.4% of the participants answered “not applicable” to the questions “Finding my car in a parking lot,” “Find it harder to remember the result of a recent sporting event,” and “Following a map to find a new location”. Some healthy older adults in Taiwan do not drive a car due to population density and convenient public transportation system with free mileage. Sports events also seemed not to be as popular as in other countries. Furthermore, the revolution of internet and prevalence of electronic appliance have changed people’s way to live. The opportunity to follow a map has become slimmer than following a dot on a mobile device. Considering the influence of cultural and generation difference, timely development of SCD questionnaire tailored for target population seemed to be important. The resultant 14-item SCDS was developed among participants speaking fluent Mandarin. Although it is plausible to be applied to people speaking other dialects, such as Taiwanese Hokkien, or Cantonese, it may require modification on the written form.

Limitations of the present study included small sample size, relatively young age of the participants and thus limited generalizability to the old-old population, lack of biomarker data and measures of personality traits, and potential sampling bias with recruitment through snowballing. The SCDS also did not include items for visuospatial function. The self-report format of the SCDS could render inaccurate results when the individual suffered from loss of insight, which is commonly seen in people with dementia of Alzheimer’s type. Hence, a caveat shall be taken with care regarding the level of awareness when using the SCDS. In addition, it remains unclear if the SCDS predicts cognitive deterioration and how well it would apply to different types of dementia. Longitudinal study is required to validate the cut-off point on SCDS for individuals with actual risk of converting to dementia.

In sum, the SCDS is a brief self-reported questionnaire that contains only 14 items but a three-factor structure. It provides an alternative approach to measure self-perceived cognitive difficulty in people with risk of subsequent deterioration. However, the scores could be affected by emotional status, such as anxiety, depression, and worries. Considering the heterogeneity of SCD, an implication of dementia risk shall only be made when the cognitive complaints are out of proportion and cannot be better explained by emotional distress.

## Supplemental Material

sj-pdf-1-aja-10.1177_15333175211038237 – Supplemental Material for Development of the Subjective Cognitive Decline Scale for Mandarin-Speaking Population Click here for additional data file. Supplemental Material, sj-pdf-1-aja-10.1177_15333175211038237 for Development of the Subjective Cognitive Decline Scale for Mandarin-Speaking Population by Hsing-Fang Tsai, Chi-Hsun Wu, Chih-Cheng Hsu, Chien-Liang Liu and Yen-Hsuan Hsu in American Journal of Alzheimer's Disease & Other Dementias
