# Supplementary material for: Development of the Subjective Cognitive Decline Scale for Mandarin-Speaking Population
Source: Am J Alzheimers Dis Other Demen. 2021 Sep 7;36:15333175211038237. doi: 10.1177/15333175211038237 (PMC10581143; doi:10.1177/15333175211038237)
Supplement: sj-pdf-1-aja-10.1177_15333175211038237 – Supplemental Material for Development of the Subjective Cognitive Decline Scale for Mandarin-Speaking Population [file sj-pdf-1-aja-10.1177_15333175211038237.pdf]

Appendix A1

台灣主觀認知功能退化量表

第一部分：評估我自己的認知功能退化情形

a. 您覺得自己有記憶或認知上的困難嗎？ 是 否

b. 在過去一年內，您的記憶或認知能力已經下降了嗎？ 是 否

c. 整體而言，您覺得您的記憶或認知能力比您同年齡的人還差嗎？ 是 否

d. 您曾經因為您的記憶或認知困難去看醫生嗎？ 是 否

e. 您會不會因為這些記憶或認知上的困難而去看醫生？ 會 不會

f. 對於您的記憶或認知問題，您的擔心程度為何？（請在下方數字圈選作答）

（不擔心）-----------（稍微擔心）----------（非常擔心）

1 2 3 4 5

第二部分：

接下來的題目會列出一系列日常生活活動。我們想瞭解您認為自己在從事以下活動的表現，與<u>**一年前**</u>相比是否有改變。請依據您對於每個題目敘述的同意程度，在每個題目右邊的數字上做圈選。

選「**1**」，表示您「**非常不同意**」此題目的描述。

選「**2**」，表示您「**不同意**」此題目的描述。

選「**3**」，表示您對此題目的描述「**中等程度同意**」。

選「**4**」，表示您「**同意**」此題目的描述。

選「**5**」，表示您「**非常同意**」此題目的描述。

若您沒有此題目所描述的活動經驗，請在「**不適用**」方格內打勾「☑」。

（請翻面繼續填寫）

	非常不同意	不同意	中等程度同意	同意	非常同意	不適用
1. 我比一年前難記住今天的日期。	1	2	3	4	5	☐
2. 我比一年前難開始從事新的或不同的事情。	1	2	3	4	5	☐
3. 原本我可以輕鬆解決的問題，現在卻變得比一年前困難。	1	2	3	4	5	☐
4. 我比一年前難一次做超過一件事情而不煩躁。	1	2	3	4	5	☐
5. 我比一年前難記住今天是星期幾。	1	2	3	4	5	☐
6. 我比一年前難記住不同事件之間的時間關聯順序。	1	2	3	4	5	☐
7. 我比一年前難找出我想要在一段對話中使用的詞彙。	1	2	3	4	5	☐
8. 我比一年前難描述出劇情。	1	2	3	4	5	☐
9. 我比一年前難一次就聽懂別人說的事情。	1	2	3	4	5	☐
10. 我比一年前難開啟一段對話。	1	2	3	4	5	☐
11. 遇到看似熟悉的人時，我比一年前容易忘記在哪裡見過他。	1	2	3	4	5	☐
12. 我和人對話時，比一年前容易忘記提到原本要說的重點。	1	2	3	4	5	☐
13. 我到店裡買東西時，比一年前容易忘記要買什麼東西。	1	2	3	4	5	☐
14. 我比一年前容易忘記自己正要去做什麼事情。	1	2	3	4	5	☐

（題目到此結束）

Translated version of the Subjective Cognitive Decline Scale (SCDS)

Part I: Evaluate my cognitive decline

a. Do you feel you have memory or cognitive difficulty? ………………………………….yes no

b. In the past one year, has your memory or cognitive ability deteriorated?.........yes no

c. In general, do you feel your memory or cognitive ability is worse than people of your age? Yes no

d. Have you visited a doctor because of these memory or cognitive difficulty? Yes no

e. Would you visit a doctor because of these memory or cognitive difficulty? Yes no

f. How worry are you concerning your memory or cognitive problem?

(not at all) (a little bit) (very much)

1 2 3 4 5

Part II

There is a list of activities of daily living below. Please rate your performance on the following activities as compared to how it was <u>one year ago</u> (1 [strongly disagreed] – 2 – 3 [somewhat agreed] – 4 – 5 [strongly agreed]). If you/they do not have experience in the activity, mark as “not applicable”.

1. Remember the present date.
2. Start new or different things.
3. Solve problems as easily as before.
4. Do more than one thing at once without getting agitated.
5. Remember what day it is.
6. Remember time relationships of different events.
7. Find the right words to use in a conversation.

8. Describe the plots of films.
9. Understand things the first time someone says them.
10. Start conversations.
11. Meet people who seem familiar but can't remember where you/they met them.
12. Forget to bring up an important point you/they had intended to mention during a conversation.
13. Forget what you/they intended to buy at a store
14. Forget what you/they were going to do.
